# Koopman–von Neumann and Weyl–Wigner Phase-Space Formulation of Inviscid Euler Flows

**DOI:** 10.3390/e28040416

**Published:** 2026-04-07

**Authors:** Sandor M. Molnar, Joseph R. Godfrey

**Affiliations:** 1 Institute of Astronomy and Astrophysics, Academia Sinica, No. 1, Section 4, Roosevelt Road, Taipei 10617, Taiwan; smmolnar@gmail.com; 2 Grado Department of Industrial and Systems Engineering, Virginia Tech, Alexandria, VA 22305, USA

**Keywords:** Koopman–von Neumann, Weyl quantization, Wigner function, Moyal bracket, Euler equation, Burgers equation, phase-space methods

## Abstract

We develop a unified Koopman–von Neumann (KvN) operator and Weyl–Wigner phase-space framework for inviscid ideal (barotropic) Euler flows. Our approach reformulates the nonlinear fluid dynamics as a linear KvN evolution on an enlarged field phase space, thereby enabling us to apply tools developed for quantum mechanics (Weyl quantization, Moyal ⋆-products, and Wigner functionals) to a classical fluid. We construct the appropriate KvN generator (including the required Jacobian term for unitarity) and derive the evolution equation for the corresponding Wigner functional. This framework clarifies when the classical Liouville (Vlasov) description is exact—namely, in quadratic or linear regimes where the Moyal bracket reduces to the Poisson bracket—and when higher-order quantum-like corrections become significant in fully nonlinear regimes. As an analytic example, we obtain a closed-form Wigner solution for a one-dimensional Burgers flow (pressureless Euler) and verify, term by term, that it reproduces the expected Liouville transport (with distributional contributions at the shock). We also compare the phase-space approach with a kinetic (Vlasov–monokinetic) formulation and outline the extension of the framework to three-dimensional flows using a Clebsch variable representation.

## 1. Introduction

Modern theoretical research has increasingly explored analogies between classical fluid dynamics and quantum mechanics. On the one hand, the Euler equations for an ideal fluid have long been known to possess a Hamiltonian structure, including Clebsch representations and non-canonical Poisson brackets. Geometric-mechanics methods have therefore been widely applied to fluid flows [1,2,3]. On the other hand, phase space quantization methods—the Weyl–Wigner–Moyal formalism—provide powerful tools in quantum mechanics for handling dynamics in terms of phase space distributions and symbolic calculus [4,5,6,7]. Our work brings these two threads together by leveraging the Koopman–von Neumann (KvN) operator formalism as a bridge.

The KvN approach recasts classical mechanics into a linear evolution of wavefunctions (or wavefunctionals) in Hilbert space, much like quantum Schrödinger dynamics but with commutative observables [8,9]. This operator-theoretic picture of classical dynamics was pioneered by Koopman and von Neumann in the 1930s [10,11], and recent developments have extended it to systems with many degrees of freedom, including continuum (field) settings and kinetic theory [12,13,14,15].

Applying Weyl quantization within this context moves us from a formal “class-ical–quantum” analogy to a quantum field-theoretic phase space formulation of the fluid dynamics. In other words, the classical Euler system (expressed in KvN form) is described using quantum-like operators together with a Wigner functional (a field phase space distribution; see, e.g., [16,17,18]). (The precise quantization of the Euler equation in this manner involves subtleties and remains an area of active research.) The *Weyl quantization* is a widely used procedure for associating a classical phase space observable with a self-adjoint operator on a Hilbert space. This rule—introduced by H. Weyl in 1927—follows symmetric operator ordering and provides a systematic route to quantization [19]. However, as Groenewold showed, no single quantization scheme can retain all the properties of classical observables [20]. In practice, the product of functions must be deformed: in the Weyl–Wigner formalism, the composition of operators corresponds to the noncommutative Groenewold–Moyal ⋆-product on phase space functions [5,20] (a cornerstone of deformation quantization [17,18]).

The *inverse Weyl transform* takes an operator to its phase space *Weyl symbol*. In particular, applying this inverse to the quantum density operator yields the Wigner quasi-probability distribution on phase space (the *Wigner function)* [4,6]. Under appropriate conditions (e.g., for trace-class or Hilbert–Schmidt operators and the corresponding square-integrable symbols), the Weyl and inverse-Weyl maps are invertible, establishing a one-to-one correspondence between operators and phase space symbols [21,22]. In this sense, one can reformulate the dynamics entirely in phase space terms: quantum-mechanical evolution can be expressed as an evolution equation for the Wigner function(al), and operator commutators are represented by Moyal brackets (the phase space analog of Poisson brackets) [5,7]. Table 1 summarizes these correspondences.

In parallel to these developments, phase-space techniques (Wigner functions and Moyal brackets) have been used to study the quantum–classical interface and have also motivated quantum/quantum-inspired computational approaches to fluid dynamics [23,24]. However, prior to our work, no study had explicitly formulated the Euler fluid equations in KvN Hilbert-space form *and* derived the corresponding Wigner phase space evolution at the level of a Wigner functional. The closest efforts either treated kinetic models (notably Vlasov-type dynamics) with Koopman/Clebsch methods [13] or focused on quantum simulations of fluid equations without developing the underlying KvN/Weyl–Wigner operator structure [23,24].

The primary motivation for this approach recently is computational: the linear, unitary nature of the KvN evolution equation is well suited for quantum computing algorithms potentially providing memory and computational advantages on quantum hardware. By representing the classical fluid state in a Hilbert space and using the KvN Hamiltonian (which is first order in derivatives, unlike the second-order Schrödinger Hamiltonian), researchers aim to leverage the potential memory and computational advantages of quantum computers to simulate complex classical fluid dynamics problems, especially those involving uncertainty quantification or turbulent flows. In quantum computing contexts, the term “KvN/Weyl quantization” of the Euler equations refers to a method using the KvN Hilbert space formulation and Weyl correspondence rules to reformulate the classical Euler equations suitable for quantum information processing and simulation.

In this paper, we develop a unified KvN–Wigner framework for the inviscid barotropic Euler equations. Building on the insights of Koopman and von Neumann [10,11] and modern KvN/Hamiltonian developments [8,9,12], we derive an explicit Koopman operator (Liouvillian) for Euler flow on an infinite-dimensional phase space of field variables. This yields a linear Schrödinger-like equation governing the fluid state—a formulation that, to our knowledge, has not appeared in the previous literature. Crucially, we identify and include the required Jacobian (functional divergence) term in the generator to ensure that the KvN evolution is unitary (probability-conserving) in the function space [12,15]. With this operator in hand, we then perform a Weyl quantization of the classical fluid variables, moving to a Wigner-functional description of the flow. Note that we introduce *ℏ* only as a formal bookkeeping parameter (a deformation parameter) rather than the physical Planck’s constant, i.e., no actual quantum fluid is being described (no physical quantization of the fluid is implied).

In regimes where the effective Hamiltonian is quadratic (or linear) in the relevant variables, we show that the Wigner-functional evolution reduces exactly to classical Liouville transport, consistent with the fact that the Moyal bracket coincides with the Poisson bracket in these cases [5,7,20,21]. Beyond this linear regime, our analysis reveals the emergence of genuine higher-order (Moyal-type) corrections in the phase space evolution. Physically, these corrections become relevant when nonlinear steepening drives the dynamics beyond a single-valued velocity description (e.g., multistream overlap in phase space).

Finally, to illustrate and validate the framework, we present a worked analytic example. We solve the 1D Burgers flow (the pressureless Euler equation) in the KvN–Wigner setting, obtaining a closed-form Wigner-functional solution that describes shock formation. This example allows us to verify, term by term, the Liouville reduction in smooth regions and to identify the contributions that account for weak/distributional behavior at kink (shock) lines [25,26,27].

We also compare our phase space approach to a kinetic (Vlasov–monokinetic) route and outline the extension to three dimensions using Clebsch potentials (also called Clebsch variables), connecting with momentum-map and Clebsch formulations in the Koopman/Vlasov setting [3,13]. Taken together, the results position our contribution at the intersection of classical hydrodynamics, operator theory, and phase space quantization, opening a pathway for applying quantum phase space tools to analyze (and potentially simulate) classical fluid phenomena in new ways.

We also compare our phase-space formulation to the standard kinetic description with a monokinetic Vlasov closure, and we outline its extension to three-dimensional flows using a Clebsch-variable formulation (connecting to known non-canonical Poisson structures [3,13]). Taken together, these developments place our contribution at the intersection of classical hydrodynamics, operator theory, and phase-space quantization. This unified perspective provides a foundation for leveraging quantum phase-space techniques in the analysis (and eventual simulation) of classical fluid phenomena.

## 2. KvN for Barotropic Euler Equation on Field Phase Space

Throughout this paper we adopt the standard Weyl–Wigner–Moyal symbol calculus and the associated Groenewold–Moyal ⋆-product as our phase space reference and convention set [4,5,6,7,20,21,28,29]. In particular, for Hamiltonians that are at most quadratic in canonical phase-space variables the Moyal bracket closes at first order in *ℏ* and coincides with the Poisson bracket, so that the Wigner evolution reduces exactly to Liouville transport. The operatorial Koopman–von Neumann lift that underpins our KvN→Weyl pipeline is standard in classical mechanics and has been developed in modern operatorial treatments [8,10,11,12].

Let the fields $\left(\phi (x),\rho (x)\right)$ be canonical with

(1) $$\begin{array}{cc} \left\{\phi (x),\rho (y)\right\} & =\delta (x-y),\\ u(x,t) & =\partial_{x}\phi \left(x,t\right), \end{array}$$

(canonical Hamiltonian fluid structure; see [30]). For a barotropic internal energy density, ε(ρ), under isentropic processes, the pressure p(ρ)=ρdε(ρ)/dρ−ε(ρ). For the Hamiltonian, we adopt

(2) $$H\left[\rho ,\phi \right]=\int dx\left[\frac{1}{2}\;\rho \left(x\right)\;{\left(\partial_{x}\phi \left(x\right)\right)}^{2}+\varepsilon \left(\rho (x)\right)\right].$$

Hamilton’s equations give the 1D compressible Euler equations:

(3) $$\begin{array}{cc} \partial_{t}\rho +\partial_{x}\left(\rho u\right) & =0,\\ \partial_{t}u+u\;\partial_{x}u+\frac{1}{\rho }\partial_{x}p\left(\rho \right) & =0. \end{array}$$

Equivalently, with $u=\partial_{x}\phi$,

(4) $$\begin{array}{cc} \partial_{t}\phi +\frac{1}{2}\;u^{2}+h\left(\rho \right) & =0,\\ h^{\prime }\left(\rho \right) & =\frac{p^{\prime }\left(\rho \right)}{\rho }. \end{array}$$

### KvN Lift and Generator

Introduce functional conjugates acting on wavefunctionals Ψ[ϕ,ρ;t]:

(5) $$\begin{array}{cc} {\hat{\lambda }}_{\phi }\left(x\right) & =-i\hbar \;\frac{\delta }{\delta \phi (x)},\\ {\hat{\lambda }}_{\rho }\left(x\right) & =-i\hbar \;\frac{\delta }{\delta \rho (x)}, \end{array}$$

where that ϕ(x) is the velocity potential and ρ(x) the density field. The classical Liouvillian symbol in this case can be expressed as

(6) $$L\left[\rho ,\phi \right]=\int dx\;\left[\;\frac{\delta H}{\delta \rho (x)}\;\lambda_{\phi }\left(x\right)\;-\;\frac{\delta H}{\delta \phi (x)}\;\lambda_{\rho }\left(x\right)\;\right].$$

Weyl symmetrizing (for unitarity) yields the KvN/Schrödinger equation

(7) $$i\hbar \;\partial_{t}=\hat{L}\;\Psi ,$$

where

(8) $$\hat{L}=\int dx\left[\frac{\delta H}{\delta \rho }\;{\hat{\lambda }}_{\phi }-\frac{\delta H}{\delta \phi }\;{\hat{\lambda }}_{\rho }\right]+\frac{i\hbar }{2}\;\mathrm{div}\;X_{H},$$

$X_{H}$ is the Hamiltonian vector field on (ϕ,ρ), and the final term is the functional divergence. This divergence (Jacobian) term is required to ensure that $\hat{L}$ is anti-Hermitian (so that ${\left|\Psi \right|}^{2}$ is conserved in time) [12]. Thus, for the Hamiltonian, Equation (2), we obtain

(9) $$\begin{array}{cc} \frac{\delta H}{\delta \rho } & =\frac{1}{2}\;u^{2}+h\left(\rho \right),\\ \frac{\delta H}{\delta \phi } & =-\;\partial_{x}\left(\rho \;u\right). \end{array}$$

As a consequence, $\hat{L}$ can be expressed as

(10) $$\hat{L}\;=\;\int dx\;\left[\left(\frac{1}{2}\;u^{2}+h\left(\rho \right)\right){\hat{\lambda }}_{\phi }\;+\;\partial_{x}\left(\rho \;u\right)\;{\hat{\lambda }}_{\rho }\right]\;+\;\frac{i\hbar }{2}\;\mathrm{div}\;X_{H}.$$

## 3. Wigner Functional and the Moyal vs. Poisson Brackets

In an infinite-dimensional (classical-field) phase space with canonical fields (ϕ(x),π(x)), the finite-dimensional Wigner function [4] extends to a *Wigner functional* W[ϕ,π;t] defined as the functional Fourier transform of the field-density matrix in the relative coordinate, providing the Weyl symbol of operators and the generator of symmetrically ordered field correlators. This construction follows the Weyl quantization map [19] and its phase space realization via the Groenewold–Moyal ⋆-product [5,20], yielding a *functional* Moyal bracket whose ℏ→0 limit reduces to the classical field-theoretic Poisson bracket. In practice, field-theoretic Wigner functionals require care with operator-valued distributions and ultraviolet regularization (and renormalization) when taking coincident-point limits [31,32]. Moreover, the functional ⋆-product is intrinsically nonlocal, so integrations by parts in field space can generate boundary terms unless suitable decay/constraint conditions are imposed [33,34].

The Euler–Poincaré equations and semidirect products with applications to continuum theories establishes the Hamiltonian/Lie–Poisson framework [3]. Poisson brackets for fluids and plasmas establishes the Hamiltonian/Lie–Poisson framework used for our Euler-side generators [1,35]. Here we follow Polkovnikov’s approach to Wigner functionals and the Moyal/Poisson brackets [16].

Let the KvN density operator, ${\hat{\rho }}_{\mathrm{KvN}}\left(t\right)$ for a pure state be

(11) $${\hat{\rho }}_{\mathrm{KvN}}\left(t\right)=|\Psi (t)\rangle \langle \Psi (t)|,$$

and

(12) $$\rho_{\mathrm{KvN}}\left[\phi_{-},\phi_{+};t\right]=\langle \phi_{-}|{\hat{\rho }}_{\mathrm{KvN}}\left(t\right)|\phi_{+}\rangle =\int D\rho \;\Psi \left[\phi_{-},\rho ;t\right]\;\Psi^{*}\left[\phi_{+},\rho ;t\right].$$

Here ∫Dρ denotes a formal functional integral over the field ρ(x). Concretely, one may define it by discretizing $x\mapsto x_{j}$ on a lattice and writing $D\rho :=\lim_{N\to \infty }\prod_{j=1}^{N}d\rho_{j}$ (and similarly for Dϕ and Dη). This functional integration measure should not be confused with the bidifferential operator $D^{\left(2n+1\right)}\left(H,W\right)$ defined in Appendix A.

We define the Wigner functional, W[ϕ,ρ;t], (the functional Weyl transformation of the KvN density operator), as

(13) $$W\left[\phi ,\rho ;t\right]\;=\;\frac{1}{N}\;\int D\eta \;\exp\;\left(\frac{i}{\hbar }\int dx\;\rho \left(x\right)\;\eta \left(x\right)\right)\;\rho_{\mathrm{KvN}}\;\left[\phi -\frac{\eta }{2},\;\phi +\frac{\eta }{2};\;t\right],$$

The evolution of the Wigner functional obeys the functional Moyal time evolution equation (also called Wigner equation),

(14) $$\partial_{t}W={\left\{H,W\right\}}_{\mathrm{PB}}+\sum_{n\geq 1}\frac{{\left(i\hbar /2\right)}^{2n}}{(2n+1)!}\;D^{\left(2n+1\right)}\left(H,W\right),$$

where ${\left\{\;\cdot ,\cdot \;\right\}}_{\mathrm{PB}}$ is the canonical functional Poisson bracket in (ϕ,ρ) and $D^{\left(k\right)}$ is the standard bidifferential operator built from *k*th functional derivatives (in our case, k=(2n+1); see Appendix A) [5,36].

When the Hamiltonian is quadratic (e.g., linear acoustics around a uniform state), all higher terms in the Moyal bracket vanish, and thus the Moyal bracket reduces to the Poisson bracket, and we obtain

(15) $$\partial_{t}W={\left\{H,W\right\}}_{\mathrm{PB}}$$

exactly. With the cubic term $\rho \;{\left(\partial_{x}\phi \right)}^{2}$ in Equation (2), $O(\hbar^{2})$ terms in Equation (14) are generically nonzero.

## 4. Worked 1D Example: Pressureless Limit, Burgers’ Equation

This example illustrates how the Wigner formalism handles a developing shock: it confirms that in smooth regions the classical Liouville limit is recovered, and it exposes where non-classical (Moyal) terms become important (at the shock discontinuity). The weak-solution and shock framework applied in our Burger’s equation example is summarized in [25].

We now present a fully explicit Wigner solution for the pressureless case, i.e., p≡0 so that u(x,t) obeys the inviscid Burgers equation. For 0<t<1, adopt the following solution, a piecewise triangular profile:

(16) $$u\left(x,t\right)=\left\{\begin{array}{cc} \frac{1+x}{1+t}, & -1<x<t,\\ \frac{1-x}{1-t}, & t<x<1,\\ 0, & \mathrm{otherwise}. \end{array}\right.$$

### 4.1. Derivation of the Wigner Function, Explicit $W_{\psi }$ Formulas

In our case, the Wigner functional (Equation (13) simplifies to the usual Wigner function. We apply Equation (13) to one degree of freedom and identify the functional variables with the single-particle ones: ϕ↦x, ρ↦p, and the functional shift η(·)↦yδ(·−x). With the usual normalization N=2π, this gives exactly the *x*–*p* Wigner transform, and using the signal-kernel convention, it can be expressed as

(17) $$W_{\psi }\left(x,p,t\right)=\int_{R}e^{ipy}\;\psi \;\left(x+\frac{y}{2},t\right)\psi^{*}\;\left(x-\frac{y}{2},t\right)\;dy.$$

This approach provides a clean derivation from the KvN/Weyl framework to the Wigner function associated with our solution to Burgers’ equation (Equation (16)).

From our solution, u(x,t), Equation (16), we obtain the phase potential, S(x,t). Along KvN characteristics, a scalar wavefunction ψ(x,t) obeys pure advection with a Jacobian factor, and thus locally one may write $\psi \left(x,t\right)=A\left(x,t\right)\;e^{iS(x,t)}$ with $\partial_{x}S\left(x,t\right)=u\left(x,t\right)$ and smooth amplitude A(x,t). For our piecewise velocity, Equation (16), integrating $\partial_{x}S_{\pm }=u$ gives the phases of the two branches, $S_{-}\left(x,t\right)$, $S_{+}\left(x,t\right)$, as

(18) $$\begin{array}{cc} S_{-}\left(x,t\right) & =\frac{x+\frac{1}{2}x^{2}}{1+t}\;\;-1<x<t,\\ S_{+}\left(x,t\right) & =\frac{x-\frac{1}{2}x^{2}}{1-t}\;\;t<x<1, \end{array}$$

up to *t*-dependent additive constants that *cancel in phase differences* (see Equation (17)) and therefore do not affect $W_{\psi }$ (only the relative phase is consequential; see Appendix B).

Using the phase functions, Equation (18), and handling the limits of the integrals with care, we obtain the Wigner function as

(19) $$W_{\psi }\left(x,p,t\right)=W_{\psi }^{\left(L\right)}\left(x,p,t\right)+W_{\psi }^{\left(R\right)}\left(x,p,t\right)+W_{\psi }^{\left(\mathrm{mix}\right)}\left(x,p,t\right),$$

where $W_{\psi }^{\left(L,R\right)}$ and mixed $W_{\psi }^{\left(\mathrm{mix}\right)}$ are the same side and mixed terms contributions coming from our piece wise linear solution (Equatoin (16)). The derivation of these terms can be found in Appendix C (Equations (A19), (A20) and (A25)).

Note, if $S_{\pm }\mapsto S_{\pm }+C_{\pm }\left(t\right)$, then every occurrence of $S\left(x+\frac{y}{2}\right)-S\left(x-\frac{y}{2}\right)$ is unchanged; hence the integrands in Equations (A19), (A20) and (A25) are invariant. Similarly, a slowly varying amplitude *A* that is identical on both sides to leading order factors out and only affects an overall prefactor, which we set to one in our normalization. For our analytic Burgers benchmark we choose unit-amplitude branches, $\psi_{R,L}\left(x,t\right)=\exp\left(iS_{R,L}\left(x,t\right)\right)$ (i.e., $A_{R,L}=1$), so that the closed-form Wigner calculation isolates the phase/interference structure without introducing additional modeling choices for branch weights via amplitude transport. More generally, a smooth amplitude would enter as prefactors $|A_{R,L}{\left(x,t\right)|}^{2}$ for same-side terms and $A_{R}A_{L}$ for mixed-side terms at leading order.

### 4.2. Physical Interpretation of the Wigner Function, $W_{\psi }$

At t→1, our solution to Burgers equation develops a shock at x=1 (e.g., [26]). In the pre-shock regime, the two Wigner “branches” correspond to the two classical characteristics approaching the forming shock. The Fresnel cross term encodes their phase-coherent interference; away from the overlap region it averages out (Riemann–Lebesgue), while near the kink it controls the weak limit used below. Note that in this classical context, the Wigner function is always non-negative, thus no negative probabilities are generated.

### 4.3. Derivation of the Pressureless/KvN Lift Hamiltonian

We show that the Koopman generator is the Weyl quantization of the classical symbol H(x,p,t)=pu(x,t), that we interpret as the Hamiltonian of the pressureless/KvN lift.

Let $\hat{x}$ denote multiplication by *x* and $\hat{p}=-\;i\;\partial_{x}$, and recall the Weyl map ${\mathrm{Op}}_{W}\left[\cdot \right]$:

(20) $$\left({\mathrm{Op}}_{W}\left[a\right]\psi \right)\left(x\right)\;=\;\frac{1}{2\pi }\;\int_{R}\;\;\int_{R}e^{\;ip(x-y)}\;a\;\left(\frac{x+y}{2},p\right)\;\psi \left(y\right)\;dp\;dy,$$

compatible with $\hat{p}=-\;i\;\partial_{x}$. Linearity in *a* allows us to expand u(x,t) as $u\left(x,t\right)=\frac{1}{2\pi }\int \hat{u}\left(k,t\right)\;e^{ikx}\;dk$. We then write the operator in Fourier space and apply the Weyl map using Equation (20). A direct calculation gives the symmetrized form. After inverse Fourier transformation, this is the standard Koopman (Liouville) generator on $L^{2}\left(R,dx\right)$ under suitable boundary conditions.

(21) $${\mathrm{Op}}_{W}\;\left[p\;u(x,t)\right]\;=\;\frac{1}{2\pi }\int \hat{u}\left(k,t\right)\;{\mathrm{Op}}_{W}\;\left[p\;e^{ikx}\right]\;dk.$$

A direct calculation using Equation (20) gives the symmetrized form

(22) $${\mathrm{Op}}_{W}\left[p\;e^{ikx}\right]\;=\;\frac{1}{2}\left(e^{ik\hat{x}}\;\hat{p}+\hat{p}\;e^{ik\hat{x}}\right),$$

and, after inverse Fourier transformation,

(23) $$\hat{L}\left(t\right)\;:=\;{\mathrm{Op}}_{W}\;\left[p\;u(x,t)\right]\;=\;\frac{1}{2}\left(u\left(\hat{x},t\right)\;\hat{p}+\hat{p}\;u\left(\hat{x},t\right)\right)\;=\;-\;i\left(u\;\partial_{x}+\frac{1}{2}\;\partial_{x}u\right),$$

which is the standard Koopman (Liouville) generator on $L^{2}\left(R,dx\right)$ under suitable boundary conditions.

In the Koopman/Weyl lift, the phase-space Hamiltonian is, by definition, the Weyl symbol of the generator: $H:={\mathrm{symb}}_{W}\left(\hat{L}\right)=L_{W}\left(x,p,t\right)$. For $\hat{L}=\frac{1}{2}\left(u\left(\hat{x},t\right)\hat{p}+\hat{p}\;u\left(\hat{x},t\right)\right)$ we have, by the Groenewold rule,

(24) $$L_{W}\left(x,p,t\right)\;=\;\frac{1}{2}\left(u⋆p+p⋆u\right)\;=\;u\;p\;=:\;H\left(x,p,t\right),$$

since $u⋆p=up+\frac{i}{2}\;\partial_{x}u$ and $p⋆u=up-\frac{i}{2}\;\partial_{x}u$, whose average cancels the ordering terms.

Equivalently, *H* is characterized (up to an additive function of *t*) by reproducing the Liouville vector field via Hamilton’s equations:

(25) $$\begin{array}{cc} \dot{x} & =\partial_{p}H\;=\;u\left(x,t\right),\\ \dot{p} & =-\;\partial_{x}H\;=\;-\;p\;\partial_{x}u\left(x,t\right). \end{array}$$

The unique solution of Equation (25) is H(x,p,t)=u(x,t)p+C(t); the additive C(t) drops out of the (Moyal/Poisson) bracket and is physically irrelevant. Thus we identify H=up.

### 4.4. Liouville Transport and Cancellation

We now verify Liouville transport equation. With our signal–Wigner convention (ℏ=1), the von Neumann equation $i\;\partial_{t}\psi =\hat{L}\left(t\right)\psi$ for $\hat{\rho }=\left|\psi \rangle \langle \psi \right|$ Weyl transforms to the Moyal evolution equation,

(26) $$\partial_{t}W_{\psi }\;=\;{\left\{H,W_{\psi }\right\}}_{⋆}\;=\;2H\;\sin\;\left(\frac{1}{2}\Lambda \right)\;W_{\psi },$$

with the bidifferential operator $\Lambda ={\overset{\leftarrow }{\partial }}_{x}\;{\vec{\partial }}_{p}-{\overset{\leftarrow }{\partial }}_{p}\;{\vec{\partial }}_{x}$. Since our Hamiltonian H(x,p,t)=pu(x,t) (pressureless/KvN lift) is linear in *p*, away from kink lines only the first (Poisson) term survives. Thus the Moyal time evolution equation reduces to Liouville transport equation,

(27) $$\partial_{t}W+u\;\partial_{x}W-\left(\partial_{x}u\right)\;p\;\partial_{p}W=0,$$

This is the Moyal bracket−Poisson bracket identity for Hamiltonians linear in *p* [5,36]. Acting on Equation (A19) with the Liouville operator $L:=\partial_{t}+u\partial_{x}-\left(\partial_{x}u\right)\;p\;\partial_{p}$ and using

(28) $$\begin{array}{c} Lu=0,\\ L\beta_{R}=-p\;\partial_{x}u,\\ LY_{R}=Y_{R}\;\partial_{x}u, \end{array}$$

we obtain the residual,

(29) $$LW_{\psi }^{\left(\mathrm{same},R\right)}=2\;\partial_{x}u\;\left[\;p\;\frac{\sin(\beta_{R}Y_{R})}{\beta_{R}^{2}}-\frac{pY_{R}}{\beta_{R}}\cos\left(\beta_{R}Y_{R}\right)+Y_{R}\cos\left(\beta_{R}Y_{R}\right)\right].$$

The left term has the analogous form with $\left(\beta_{L},Y_{L}\right)$:

(30) $$LW_{\psi }^{\left(\mathrm{same},L\right)}=2\;\partial_{x}u\;\left[\;p\;\frac{\sin(\beta_{L}Y_{L})}{\beta_{L}^{2}}-\frac{pY_{L}}{\beta_{L}}\cos\left(\beta_{L}Y_{L}\right)+Y_{L}\cos\left(\beta_{L}Y_{L}\right)\right].$$

For the mixed term Equation (A25), differentiating C,S via Equation (A26) and using the chain rule for $w_{k}=w\left(y_{k}\right)$ yields (after cancellation) the exact opposite:

(31) $$\begin{array}{cc} LW_{\psi }^{\left(\mathrm{mix}\right)} & =-2\;\partial_{x}u\;[\;\left(p\;\frac{\sin(\beta_{R}Y_{R})}{\beta_{R}^{2}}-\frac{pY_{R}}{\beta_{R}}\cos\left(\beta_{R}Y_{R}\right)+Y_{R}\cos\left(\beta_{R}Y_{R}\right)\right)1_{\left(t,1\right)}\left(x\right)+\\ \; & \left(p\;\frac{\sin(\beta_{L}Y_{L})}{\beta_{L}^{2}}-\frac{pY_{L}}{\beta_{L}}\cos\left(\beta_{L}Y_{L}\right)+Y_{L}\cos\left(\beta_{L}Y_{L}\right)\right)1_{\left(-1,t\right)}\left(x\right)\;]. \end{array}$$

Adding Equations (29)–(31) gives $LW_{\psi }=0$ for x≠±1 and x≠t, i.e., Equation (27). Thus, for this example, we explicitly see that *W* evolves according to the classical Liouville equation in smooth regions (⋆-corrections cancel out), with delta-function contributions appearing exactly at the shock to balance the transport equation-consistent with the weak solution.

### 4.5. Weak Formulation at Kink Lines

At the kink lines x=±1 and x=t, where our solution to Burgers equation, u(x,t) (Equation (16)), is non-differentiable, the motion of integration limits produces delta function contributions that cancel in the weak (test-function) formulation.

Let $\varphi \in C_{c}^{\infty }\left(R_{x}\times R_{p}\times \left(0,T\right)\right)$ be a compactly supported test function. Integrating the transport equation by parts defines the weak solution:

(32) $$\;\int \int \int \;\left[W\;\partial_{t}\varphi \;+\;u\;W\;\partial_{x}\varphi \;-\;\left(\partial_{x}u\right)\;p\;W\;\partial_{p}\varphi \right]\;dp\;dx\;dt\;=\;0.$$

Across a moving kink x=ξ(t), the distributional calculus yields boundary contributions. For the two-branch+Fresnel structure used here, the branch terms match the characteristics and the oscillatory Fresnel piece is odd under the local interchange of branches; its integral against φ vanishes in the limit of vanishing smoothing (Riemann–Lebesgue integral), giving the claimed cancellation. This is the precise sense in which the Liouville transport remains valid across kink lines for H=pu(x,t) in our approach.

Let $\varphi \in C_{c}^{\infty }\left(R^{2}\times \left(0,1\right)\right)$. Integrate Equation (27) against φ and integrate by parts on each smooth subregion, tracking jumps in $Y_{L/R}$ across x=±1 and x=t. Endpoint terms produced by $\partial_{t}Y$ and $\partial_{x}Y$ cancel those coming from the mixed piece Equation (A25); thus $\langle LW_{\psi },\varphi \rangle =0$.

Beyond characteristic crossing, Burgers’ equation is interpreted in the weak sense with an admissibility (entropy) condition. We adopt the standard Lax/Oleinik entropy selection via Rankine–Hugoniot jump conditions; see, e.g., the finite-volume framework and convergence results summarized in LeVeque [25]. Our KvN/Weyl construction remains valid at the level of distributions, but it does not by itself fix the entropy selection; this must be imposed consistently with the PDE.

## 5. Kinetic (Vlasov–Monokinetic) Route and Comparison

The monokinetic route means that we start from a kinetic (phase space) description in terms of a distribution function f(t,x,v) evolving by a Vlasov-type transport equation, and then take a *monokinetic* (cold-fluid) closure in which f(t,x,v) concentrates at a single velocity, f(t,x,v)=ρ(t,x)δ(v−u(t,x)). Under this ansatz the velocity moments close (no independent pressure tensor), so the kinetic equation reduces to a pressureless (or barotropic, depending on the force closure) Euler-type system up to the onset of multistreaming/caustics (and, in 1D, up to shock formation).

Kang and Vasseur’s contribution [27] provides a rigorous prototype of this passage: it relates kinetic/Vlasov descriptions to macroscopic (aggregation / fluid-limit) equations via compactness and relative-entropy type arguments, and it makes precise in what sense *f* collapses toward a monokinetic measure in *v* in the limit. This is the specific “kinetic-to-fluid” benchmark we compare against our field-theoretic KvN/Weyl route.

Kinetic/Vlasov-to-fluid insights relevant for our comparisons also appear in [37], where Brenier studies the Vlasov–Monge–Ampère equation and exhibits solutions with *concentration* (measure-valued limits). This is relevant here because it provides an explicit setting in which a kinetic equation generates (or approximates) an Euler-type macroscopic dynamics while allowing singular limits and delta-like concentrations, highlighting the same kind of “closure vs. concentration” issues that arise in monokinetic reductions and in distributional manipulations.

To make the comparison concrete, we consider a single-particle phase space (x,v) and adopt the Hamiltonian

(33) $$\begin{array}{cc} H(x,v;t) & =\frac{v^{2}}{2}+U\left(x,t\right),\\ U_{x}\left(x,t\right) & =\partial_{x}\mu \left(\rho \left(x,t\right)\right),\\ \rho (x,t) & =\int_{R}f\left(x,v,t\right)\;dv, \end{array}$$

where μ(ρ(x,t)) is a local equation-of-state potential (chemical potential/specific enthalpy), generating the mean-field force $F=-\partial_{x}\mu \left(\rho \right)$. In the monokinetic (cold-fluid) limit this corresponds to an isentropic Euler pressure law p=p(ρ) through $\mu^{\prime }\left(\rho \right)=p^{\prime }\left(\rho \right)/\rho$ (equivalently, $\partial_{x}\mu \left(\rho \right)=\left(1/\rho \right)\;\partial_{x}p\left(\rho \right)$).

In the semiclassical comparison one may identify the kinetic density *f* with the Wigner function *W* (see, e.g., [38]), and the Vlasov equation reads

(34) $$\partial_{t}f+v\;\partial_{x}f-U_{x}\;\partial_{v}f=0.$$

This evolution is exact when *U* is at most quadratic in *x* (so that no higher-order $\hbar^{2}$ corrections appear). For a general U(x,t), it is recovered as the leading semiclassical term, with corrections involving higher *x*-derivatives of *U*.

**Figure 1:**
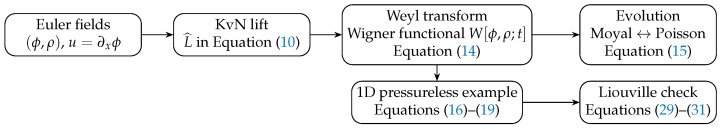
Field-theoretic pipeline: from Euler variables to the KvN generator, Weyl/Wigner functional, and evolution, with an explicit 1D worked example and Liouville verification.

**Figure 2:**
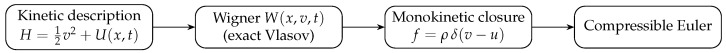
Kinetic pipeline: quadratic+potential symbol gives exact Liouville/Vlasov Wigner evolution; the monokinetic closure recovers Euler equation from Vlasov equation up to shocks.

Under the monokinetic ansatz f(t,x,v)=ρ(t,x)δ(v−u(t,x)), the zeroth and first velocity moments of (34) yield the continuity equation and a pressureless Euler momentum equation (up to shock formation), with forcing $U_{x}$ evaluated on the monokinetic state. We summarize our field-theoretic and kinetic pipelines in Figure 1 and Figure 2.

## 6. Irrotational Barotropic 3D Euler Equation as a Field Hamiltonian

We assume barotropic, compressible, *irrotational* flow with energy density local in ρ(r,t) and write the Hamiltonian as

(35) $$H\left[\rho ,\phi \right]\;\equiv \;\int_{R^{3}}d^{3}r\;\left[\frac{1}{2}\;\rho \left(r,t\right)\;{\left|\nabla \phi \left(r,t\right)\right|}^{2}+\varepsilon \;\left(\rho (r,t)\right)\right],$$

where ρ(r,t)>0 is the mass density, ϕ(r,t) is the velocity potential, and ${\left|\nabla \phi \right|}^{2}\equiv {\left(\partial_{x}\phi \right)}^{2}+{\left(\partial_{y}\phi \right)}^{2}+{\left(\partial_{z}\phi \right)}^{2}$. We use the canonical field Poisson bracket

(36) $$\left\{F,G\right\}\;=\;\int_{R^{3}}d^{3}r\;\left(\frac{\delta F}{\delta \phi }\;\frac{\delta G}{\delta \rho }-\frac{\delta F}{\delta \rho }\;\frac{\delta G}{\delta \phi }\right)\;,$$

so that the Hamiltonian evolution is $\partial_{t}F=\left\{F,H\right\}$ for any functional F[ρ,ϕ]. We work on $\Omega =R^{d}$ (or a periodic box), with data decaying sufficiently fast (or periodic) so that integrations by parts are legitimate. Pre-shock we assume ρ>0 with $\rho ,\phi \in C^{1}$ in space and time, and sufficient regularity for the canonical bracket and variational derivatives to be well defined. Boundary terms vanish under the stated conditions.

Varying Equation (35) gives

(37) $$\begin{array}{cc} \frac{\delta H}{\delta \phi } & =-\;\nabla \;\cdot \;\left(\rho \;\nabla \phi \right),\\ \frac{\delta H}{\delta \rho } & =\frac{1}{2}\;{\left|\nabla \phi \right|}^{2}+h\left(\rho \right), \end{array}$$

where $h\left(\rho \right)\equiv \varepsilon^{\prime }\left(\rho \right)$ is the specific enthalpy. Using (36) we obtain the barotropic, irrotational Euler system

(38) $$\partial_{t}\rho \;=\;\left\{\rho ,H\right\}\;=\;-\;\nabla \;\cdot \;\left(\rho \;\nabla \phi \right),$$

and

(39) $$\partial_{t}\phi \;=\;\left\{\phi ,H\right\}\;=\;-\left(\frac{1}{2}\;{\left|\nabla \phi \right|}^{2}+h\left(\rho \right)\right)\;.$$

Defining the velocity by potential flow,

(40) u(r,t)≡∇ϕ(r,t),

Equation (38) becomes the continuity equation $\partial_{t}\rho +\nabla \;\cdot \left(\rho \;u\right)=0$, while taking ∇ of (39) yields the momentum equation

(41) $$\partial_{t}u+\left(u\;\cdot \;\nabla \right)u+\nabla h\left(\rho \right)\;=\;0\;,$$

i.e., the compressible, barotropic Euler equation for irrotational flows.

For a barotropic energy density ε(ρ), one has

(42) $$\begin{array}{cc} p(\rho ) & =\rho \;\varepsilon^{\prime }\left(\rho \right)-\varepsilon \left(\rho \right),\\ \nabla h(\rho ) & =\frac{1}{\rho }\;\nabla p\left(\rho \right), \end{array}$$

so (41) can be written as $\partial_{t}u+\left(u\;\cdot \;\nabla \right)u+\rho^{-1}\nabla p\left(\rho \right)=0$.

The canonical bracket (ρ,ϕ) is equivalent to the noncanonical (Lie–Poisson/Euler–Poincaré) bracket. The equivalence between the canonical (ρ,ϕ) bracket and the compressible Euler Lie–Poisson form for (m=ρu, D=ρ) can be seen the following way. Let m=ρu denote the momentum density and D=ρ the mass density. Define functionals F[m,D] and G[m,D]. The (compressible, barotropic) Lie–Poisson bracket reads

(43) $${\left\{F,G\right\}}_{\mathrm{LP}}\;=\;-\int_{\Omega }d^{3}r\;\left[m\cdot \left(\left(\nabla F_{m}\right)\;G_{m}-\left(\nabla G_{m}\right)\;F_{m}\right)\;+\;D\;\left(F_{m}\cdot \nabla G_{D}-G_{m}\cdot \nabla F_{D}\right)\right],$$

where $F_{m}\equiv \delta F/\delta m$, $F_{D}\equiv \delta F/\delta D$, etc. With the barotropic Hamiltonian,

(44) $$H\left[m,D\right]=\int \left(\frac{1}{2}\;{\left|m\right|}^{2}/D+\varepsilon \left(D\right)\right)\;d^{3}r,$$

one obtains

(45) $$\begin{array}{cc} \partial_{t}m & ={\left\{m,H\right\}}_{\mathrm{LP}},\\ \partial_{t}D & ={\left\{D,H\right\}}_{\mathrm{LP}}, \end{array}$$

which reproduce the Euler equations in momentum form. In irrotational fluids, u=∇ϕ and m=D∇ϕ, the canonical bracket on (ρ,ϕ), Equation (36), is *equivalent* to (43) under the change of variables (ρ,ϕ)↦(m=ρ∇ϕ,D=ρ) and the Hamiltonian $H\left[\rho ,\phi \right]=\int (\frac{1}{2}{\;\rho \;|\nabla \phi |}^{2}+\varepsilon \left(\rho \right))\;d^{3}r$. A direct calculation verifies that the canonical equations, Equations (38)–(41) push forward to (45); see, e.g., [1,3,35].

### 6.1. KvN/Weyl Single–Particle Symbol and Exact Liouville Reduction

The Koopman (Liouville) generator for advection by u is $L=u\left(r,t\right)\;\cdot \;\nabla_{r}$. Its Weyl symbol is the Hamiltonian linear in momentum,

(46) H(r,p,t)=u(r,t)·p,

whose Hamilton equations are

(47) $$\begin{array}{cc} \dot{r} & =\partial_{p}H\;=\;u\left(r,t\right),\\ \dot{p} & =-\;\partial_{r}H\;=\;-\;{\left(\nabla u\right)}^{\;⊤}p. \end{array}$$

Because H is at most linear in p, the Moyal bracket reduces to the classical Poisson bracket identically, so the KvN/Weyl evolution is *exactly* Liouvillian in any dimension for (46). Assume that u(r,t) is sufficiently smooth, and consider the Weyl/Moyal evolution $\partial_{t}A={\left\{A,H\right\}}_{\mathrm{Moyal}}$ for symbols A(r,p,t) with Hamiltonian, Equation (46). Then for all such *A*,

(48) $${\left\{A,H\right\}}_{\mathrm{Moyal}}\;=\;{\left\{A,H\right\}}_{\mathrm{Poisson}}\;=\;\nabla_{r}A\cdot \nabla_{p}H\;-\;\nabla_{p}A\cdot \nabla_{r}H\;.$$

This is because the Moyal bracket admits the *ℏ*-series in bidifferential operators, and for symbols at most linear in p, all terms beyond the first order vanish identically (Groenewold’s expansion). Equivalently, using Bopp operators [16], the star-commutator with an affine symbol truncates at first order, yielding the classical Poisson bracket (see Appendix E). Rigorous statements for quadratic/affine classes can be found in [20,29,39].

### 6.2. Limitations

The field Hamiltonian (35) with the canonical bracket (36) produces the standard barotropic Euler system (38)–(41). It covers polytropic, isothermal, and general γ–law gases. The KvN/Weyl lift (46) is exact (Moyal bracket simplifies to the Poisson bracket) and cleanly separates characteristics (47) providing a clean, pedagogical KvN/Weyl lift with exact Liouville reduction via (46). This system is ideal for discussing the pressureless limit (set h≡0) and for pre-shock (single-valued) regimes.

The Hamiltonian (35) omits density-gradient terms (e.g., Korteweg/quantum stresses such as ${\left(\nabla \rho \right)}^{2}$ or ρΔlnρ). Including them modifies δH/δρ by higher-derivative contributions, and thus additional dispersive forces appear, and the simple H=u·p no longer suffices to capture the full field dynamics at the symbol level. In the Post-crossing regime, as with pressureless/Burgers, characteristic crossing requires weak/distributional solutions and appropriate entropy selections; the Hamiltonian forms remain, but the classical solution concept changes. Including vorticity would require Clebsch potentials, u=∇ϕ+α∇β, with the kinetic term $\frac{1}{2}\;\rho \;{\left|u\right|}^{2}$ and the same bracket (36).

## 7. 3D Extension, General Case: Clebsch Potentials

We can locally represent a 3D ideal flow via Clebsch potentials, e.g., u=∇ϕ+α∇β, with a barotropic Hamiltonian $H=\int \frac{1}{2}\rho {∥u∥}^{2}+\varepsilon \left(\rho \right)\;d^{3}x$ and brackets that realize the canonical structure for (ϕ,ρ) plus advected scalars (α,β). The KvN lift follows Equation (7) having applied the necessary changes, with a functional divergence term ensuring unitarity. Global/topological obstructions are gauge issues rather than algebraic ones and do not affect the local KvN/Weyl construction.

With Clebsch potentials, the canonical bracket extends to (ρ,ϕ,α,β) with the kinetic energy $\frac{1}{2}\;\rho \;{\left|\nabla \phi +\alpha \nabla \beta \right|}^{2}$. The resulting equations reproduce the full barotropic Euler system with vorticity ω=∇α×∇β. However, the Koopman/Weyl single-particle symbol H=u·p remains linear in p and exact (Moyal bracket simplifies to the Poisson bracket) *only* at the level of advection by the instantaneous u; care is required with topology/gauge when defining global phases and action functionals.

The Clebsch representation u=∇ϕ+α∇β is non-unique: (ϕ,α,β)↦(ϕ+χ(α,β),α,β) leaves u invariant up to gradients, implying a gauge freedom. On simply connected domains any irrotational field admits a single-valued ϕ, but vortical flows require multi-valued potentials or patchwise charts; vortex lines correspond to defects where (α,β) fail to be globally well defined. This has implications for boundary terms and helicity, $\int u\cdot \left(\nabla \times u\right)\;d^{3}r$, and can be expressed via Clebsch potentials only as modulo gauge corrections.

## 8. Conclusions and Outlook

We have developed a unified operator-theoretic framework for inviscid Euler and Burgers flows, bridging the Koopman–von Neumann (KvN) Hilbert-space formalism and the Weyl–Wigner–Moyal phase-space calculus. The main advantage of this approach is that the nonlinear Euler dynamics can be reformulated as a linear KvN evolution on an enlarged *field* phase space. In this formulation, familiar tools in phase space—Weyl operator symbols, ⋆-products, Wigner functionals, and systematic semiclassical expansions—become available for analyzing classical fluids.

We derived the induced evolution equation for the Wigner functional. Using Weyl symbol calculus, we made it precise when the classical Liouville transport picture is exact and when higher-order (Moyal) corrections are unavoidable. In particular, we verified that in quadratic/linear regimes (where the ⋆-commutator truncates) the Wigner evolution reduces exactly to classical Liouville transport, consistent with known results [5,20]. We also identified the fully nonlinear Euler terms that necessarily generate $O(\hbar^{2})$ corrections in the Wigner-functional evolution.

As a worked analytic benchmark, we solved the one-dimensional pressureless limit (Burgers flow) in closed form at the level of the Wigner functional. This example makes the framework concrete and yields insight into its capabilities and limits. It allows a direct, term-by-term verification of the Liouville equation, including distributional boundary terms supported on kink lines. This example demonstrates the necessity of measure-theoretic treatment (distributional contributions at kink lines) and suggests how similar issues may appear in higher dimensions. Note that our KvN–Wigner description remains valid in a distributional sense through shock formation, but it does not resolve the unique entropy-satisfying solution without an additional selection principle (we applied the standard Lax/Oleinik condition in our example).

We also compared with a kinetic (Vlasov–monokinetic) route. That route yields an exact Liouville transport equation for the Wigner density under the monokinetic closure. Finally, we outlined how the construction extends to three dimensions using Clebsch-type variables, including the associated gauge structure.

In summary, we developed a unified KvN–Wigner phase-space framework for ideal Euler flows, which to our knowledge, has not been explicitly formulated before. It enables classical fluid dynamics to be analyzed with quantum-phase-space tools.

Several extensions are natural and appear technically within reach.

(i) *General barotropic models and gradient-energy corrections:* The present formulation treats standard barotropic enthalpy consistently. It is also straightforward to incorporate additional density-gradient (Korteweg/“quantum pressure”) terms at the Hamiltonian level. These modify δH/δρ by higher-derivative contributions. They provide a clean setting to study how ⋆-corrections generate controlled “interference-like” effects in otherwise classical fluid evolution.
(ii) *Vorticity and topology:* The 3D Clebsch formulation suggests a systematic treatment of vortical flows within the same KvN/Weyl language. Global/topological issues then enter as gauge constraints. They appear as constraints rather than as obstructions to the local operator calculus.
(iii) *Shocks and weak solutions:* The Burgers benchmark indicates how distributional terms arise in the Wigner-functional evolution near kinks. Extending this analysis to shock formation and to multi-stream (non-monokinetic) regimes motivates a KvN density-operator (mixed-state) formulation. It also calls for a careful weak/measure-theoretic treatment of the resulting transport equations.
(iv) *Computation:* The linear KvN evolution and the explicit separation between Liouville and higher-order ⋆-terms suggest practical numerical strategies. These include semiclassical truncations and symbolic/FFT-based evaluation of ⋆-corrections.

They also include hybrid PDE–phase-space solvers that can be benchmarked against the closed-form 1D results presented here. In the genuinely non-quadratic regime, the Moyal bracket no longer truncates at Poisson order, so that higher-order ⋆-terms, beginning at $O(\hbar^{2})$, must, in general, be retained. A numerical comparison between truncated or fully evaluated ⋆-evolution and exact dynamics would therefore be valuable, but such an analysis lies beyond the scope of the present paper.

These directions would further clarify when classical Liouville transport is sufficient. They would also clarify when genuinely non-Poisson (Moyal) structure must be retained in phase-space descriptions of ideal fluid dynamics.

## Figures and Tables

**Table 1:** Weyl–Wigner correspondences used in this paper.

Map/Object	Meaning
Weyl quantization $f\left(x,p\right)\mapsto \hat{f}$	phase space observable → operator
Inverse Weyl $\hat{f}\mapsto f_{W}\left(x,p\right)$	Operator → Weyl symbol
Wigner transform $\hat{\rho }\mapsto W\left(x,p\right)$	Density operator → Wigner function
Star product f⋆g	Operator product in phase space
Moyal bracket ${\left\{f,g\right\}}_{⋆}$	Commutator in phase space

## Data Availability

No new data were created or analyzed in this study. Data sharing is not applicable to this article.

## Appendix A. Bidifferential Operator

$D^{\left(2n+1\right)}\left(H,W\right)$ denotes the (2n+1)–fold action of the canonical Poisson bivector on field phase space, acting as a bidifferential operator between *H* and *W*:

(A1) $$\overset{↔}{\Lambda }\;:=\;\int dx\;\left(\overset{\leftarrow }{\frac{\delta }{\delta \phi (x)}}\;\vec{\frac{\delta }{\delta \rho (x)}}-\overset{\leftarrow }{\frac{\delta }{\delta \rho (x)}}\;\vec{\frac{\delta }{\delta \phi (x)}}\right),$$

so that the canonical Poisson bracket is ${\left\{H,W\right\}}_{\mathrm{PB}}=H\;\overset{↔}{\Lambda }\;W$. The higher-order Moyal terms are generated by repeated application of $\overset{↔}{\Lambda }$:

(A2) $$D^{\left(2n+1\right)}\left(H,W\right)\;:=\;H\;{\left(\overset{↔}{\Lambda }\right)}^{2n+1}W,\;n=1,2,\ldots ,$$

equivalently,

(A3) $${\left\{H,W\right\}}_{⋆}\;=\;\frac{2}{\hbar }\;H\;\sin\;\left(\frac{\hbar }{2}\;\overset{↔}{\Lambda }\right)\;W.$$

The series expansion of Equation (A3) gives Equation (14).

As an example, for n=1, we obtain

(A4) $${\left\{H,W\right\}}_{⋆}\;=\;{\left\{H,W\right\}}_{\mathrm{PB}}\;-\;\frac{\hbar^{2}}{24}\;H\;\Lambda^{3}\;W\;+\;O\left(\hbar^{4}\right).$$

Define

(A5) $$\Lambda \;=\;A-B,\;A\;:=\;\int dx\;\overset{\leftarrow }{\frac{\delta }{\delta \phi (x)}}\;\vec{\frac{\delta }{\delta \rho (x)}},\;B\;:=\;\int dx\;\overset{\leftarrow }{\frac{\delta }{\delta \rho (x)}}\;\vec{\frac{\delta }{\delta \phi (x)}}.$$

The explicit cubic bidifferential term becomes

(A6) $$D^{\left(3\right)}\left(H,W\right)\;:=\;H\;\Lambda^{3}\;W\;=\;H\;\left(A^{3}-3A^{2}B+3AB^{2}-B^{3}\right)\;W.$$

Thus, we obtain the fully expanded triple-functional-derivative form

(A7) $$\begin{array}{cc} HA^{3}W\; & =\;\int \int \int \;dx_{1}\;dx_{2}\;dx_{3}\;\overset{\leftarrow }{\frac{\delta^{3}H}{\delta \phi_{1}\;\delta \phi_{2}\;\delta \phi_{3}}}\;\vec{\frac{\delta^{3}W}{\delta \rho_{1}\;\delta \rho_{2}\;\delta \rho_{3}}},\\ HA^{2}B\;W\; & =\;\int \int \int \;dx_{1}\;dx_{2}\;dx_{3}\;\overset{\leftarrow }{\frac{\delta^{3}H}{\delta \phi_{1}\;\delta \phi_{2}\;\delta \rho_{3}}}\;\vec{\frac{\delta^{3}W}{\delta \rho_{1}\;\delta \rho_{2}\;\delta \phi_{3}}},\\ HAB^{2}W\; & =\;\int \int \int \;dx_{1}\;dx_{2}\;dx_{3}\;\overset{\leftarrow }{\frac{\delta^{3}H}{\delta \phi_{1}\;\delta \rho_{2}\;\delta \rho_{3}}}\;\vec{\frac{\delta^{3}W}{\delta \rho_{1}\;\delta \phi_{2}\;\delta \phi_{3}}},\\ HB^{3}W\; & =\;\int \int \int \;dx_{1}\;dx_{2}\;dx_{3}\;\overset{\leftarrow }{\frac{\delta^{3}H}{\delta \rho_{1}\;\delta \rho_{2}\;\delta \rho_{3}}}\;\vec{\frac{\delta^{3}W}{\delta \phi_{1}\;\delta \phi_{2}\;\delta \phi_{3}}}, \end{array}$$

where $\phi_{i}\;=\;\phi \left(x_{i}\right)$ and $\rho_{i}\;=\;\rho \left(x_{i}\right)$.

## Appendix B. Deriving the Branch Phases *S*_−_ (*x*,*t*), *S*_+_ (*x*,*t*) from *u*(*x*,*t*)

We recall the piecewise velocity u(x,t) from Equation (16). The branch phases $S_{\pm }\left(x,t\right)$ are defined as spatial primitive functions (antiderivatives) of *u* on each side of the interface (so that only phase *differences* enter the Wigner kernel, and any purely *t*-dependent constants can be dropped):

(A8) $$\partial_{x}S_{-}\left(x,t\right)\;=\;\frac{1+x}{\;1+t\;}\;,\;-1<x<t\;;$$

and

(A9) $$\partial_{x}S_{+}\left(x,t\right)\;=\;\frac{1-x}{\;1-t\;}\;,\;t<x<1\;.$$

Integrating (A8) and (A9) with respect to *x* gives

(A10) $$S_{-}\left(x,t\right)\;=\;\int \frac{1+x}{1+t}\;dx\;=\;\frac{x+\frac{1}{2}x^{2}}{\;1+t\;}\;+\;C_{-}\left(t\right)\;,\;-1<x<t\;,$$

and

(A11) $$S_{+}\left(x,t\right)\;=\;\int \frac{1-x}{1-t}\;dx\;=\;\frac{x-\frac{1}{2}x^{2}}{\;1-t\;}\;+\;C_{+}\left(t\right)\;,\;t<x<1\;.$$

Because the Wigner phase always appears as a *difference* $S\;\left(x+\frac{y}{2},t\right)-S\;\left(x-\frac{y}{2},t\right)$, the additive functions $C_{\pm }\left(t\right)$ cancel and can be set to zero without loss of generality. Thus the explicit branch phases used in Equation (16) are

(A12) $$S_{-}\left(x,t\right)\;=\;\frac{x+\frac{1}{2}x^{2}}{\;1+t\;}\;,\;-1<x<t\;;$$

(A13) $$S_{+}\left(x,t\right)\;=\;\frac{x-\frac{1}{2}x^{2}}{\;1-t\;}\;,\;t<x<1\;.$$

For later use in the same-side Wigner contributions, it is convenient to record the corresponding phase differences (linear in the separation *y* because $S_{\pm }$ are quadratic in *x*):

(A14) $$S_{-}\;\left(x+\frac{y}{2},t\right)\;-\;S_{-}\;\left(x-\frac{y}{2},t\right)\;=\;\frac{y\;\left(1+x\right)}{\;1+t\;}\;,$$

(A15) $$S_{+}\;\left(x+\frac{y}{2},t\right)\;-\;S_{+}\;\left(x-\frac{y}{2},t\right)\;=\;\frac{y\;\left(1-x\right)}{\;1-t\;}\;.$$

*Consistency checks.* Differentiating (A12) and (A13) in *x* recovers the left/right branches of u(x,t) in Equation (16). If desired, the functions $C_{\pm }\left(t\right)$ may be chosen to make *S* continuous across x=t; this choice does not affect the Wigner phase differences (A14) and (A15).

## Appendix C. Derivation of the Terms of the Wigner Function

First we derive the pure same-side contributions. Right branch (t<x<1) admits x±y/2∈(t,1), i.e., $I_{R}\left(x,t\right)=\left(-Y_{R},Y_{R}\right)$ with

(A16) $$\begin{array}{cc} Y_{R}\left(x,t\right) & =2\;\min\{\;x-t,\;1-x\;\},\\ \beta_{R}\left(x,p,t\right) & =p+\frac{1-x}{1-t}. \end{array}$$

Left branch (−1<x<t) admits x±y/2∈(−1,t), i.e., $I_{L}\left(x,t\right)=\left(-Y_{L},Y_{L}\right)$ with

(A17) $$\begin{array}{cc} Y_{L}\left(x,t\right) & =2\;\min\{\;x+1,\;t-x\;\},\\ \beta_{L}\left(x,p,t\right) & =p+\frac{1+x}{1+t}, \end{array}$$

Since the linear phase differences are

(A18) $$\begin{array}{cc} S_{+}\left(x+\frac{y}{2}\right)-S_{+}\left(x-\frac{y}{2}\right) & =\frac{y(1-x)}{1-t},\\ S_{-}\;\left(x+\frac{y}{2},t\right)-S_{-}\;\left(x-\frac{y}{2},t\right) & =\frac{y(1+x)}{1+t}. \end{array}$$

Substituting Equations (A18) into the Wigner integral (Equation (17)) and restricting the *y*-integration to admissible windows $I_{R}\left(x,t\right)=\left(-Y_{R},Y_{R}\right)$ and $I_{L}\left(x,t\right)=\left(-Y_{L},Y_{L}\right)$ yields the elementary integrals for the Wigner functions,

(A19) $$W_{\psi }^{\left(R\right)}\left(x,p,t\right)=\int_{-Y_{R}}^{Y_{R}}e^{i\beta_{R}y}\;dy=\frac{2\;\sin\;\left(\beta_{R}\;Y_{R}\right)}{\beta_{R}}.$$

and

(A20) $$W_{\psi }^{\left(L\right)}\left(x,p,t\right)=\frac{2\;\sin\;\left(\beta_{L}\;Y_{L}\right)}{\beta_{L}}.$$

Mixed contributions occur only when the *y*-interval straddles x=t,

(A21) |x−t|<min{1−x,x+1}⟺|x|+|x−t|<1.

In this case, the phase is *piecewise* $S_{-}$ on one side and $S_{+}$ on the other. The total phase difference becomes a *quadratic* polynomial in *y*. The admissible window becomes

(A22) $$\begin{array}{cc} y & \in \left[y_{0}\left(x,t\right),\;y_{1}\left(x\right)\right],\\ y_{0} & =2\;|x-t|,\\ y_{1} & =2\;\min\{\;1-x,\;x+1\;\}. \end{array}$$

After assembling the two halves, completing the square gives

(A23) $$\begin{array}{cc} \Phi (y) & =D\;y^{2}+B\;y+\Gamma ,\\ D & =\frac{1}{4\;(t^{2}-1)},\\ B & =p+\frac{t\;x-1}{t^{2}-1},\\ \Gamma & =\frac{x^{2}-2tx}{t^{2}-1}, \end{array}$$

on the restricted interval $y\in [y_{0}\left(x,t\right),y_{1}\left(x\right)]$ from Equation (A22). With the shift $y\mapsto y+\frac{B}{2D}$ and the Fresnel scalings,

(A24) $$w_{k}=w\left(y_{k}\right):=\sqrt{\frac{2\;|D|}{\pi }}\left(y_{k}+\frac{B}{2D}\right)=\frac{\;y_{k}-2\left(1-t^{2}\right)\;p+2\left(tx-1\right)\;}{\sqrt{2\pi (1-t^{2})}},\;k\in \left\{0,1\right\},$$

$\Delta C:=C\left(w_{1}\right)-C\left(w_{0}\right)$ and $\Delta S:=S\left(w_{1}\right)-S\left(w_{0}\right)$, where C(z) and S(z) are the Fresnel integrals (see Appendix D), thus the mixed integral reduces to standard Fresnel jumps, and we obtain

(A25) $$W_{\psi }^{\left(\mathrm{mix}\right)}\left(x,p,t\right)=\sqrt{\frac{2\pi }{\left|D\right|}}\;\left[\;\Delta C\;\cos\Phi \;+\;\Delta S\;\sin\Phi \;\right],$$

on the domain in Equation (A21), and $W_{\psi }^{\left(\mathrm{mix}\right)}=0$ otherwise. Checking limits: as $\beta_{L/R}\to 0$, each same-side term has a removable limit $W\to 2Y_{L/R}\left(x,t\right)$.

## Appendix D. Fresnel Integrals and Identities

Fresnel integrals are defined as

(A26) $$\begin{array}{cc} C(z) & =\int_{0}^{z}\cos\;\left(\frac{\pi }{2}\tau^{2}\right)d\tau ,\\ S(z) & =\int_{0}^{z}\sin\;\left(\frac{\pi }{2}\tau^{2}\right)d\tau , \end{array}$$

so that $C^{\prime }\left(z\right)=\cos\;\left(\frac{\pi }{2}z^{2}\right)$ and $S^{\prime }\left(z\right)=\sin\;\left(\frac{\pi }{2}z^{2}\right)$. For an indicator, we write $1_{I}\left(x\right)$.

Differentiating Equation (A24) and using Equation (A26) gives

(A27) $$\begin{array}{cc} \partial_{q}\Delta C & =c_{1}\;\partial_{q}w_{1}-c_{0}\;\partial_{q}w_{0},\\ \partial_{q}\Delta S & =s_{1}\;\partial_{q}w_{1}-s_{0}\;\partial_{q}w_{0}, \end{array}$$

where q∈{x,p,t}, $c_{k}=\cos\;\left(\frac{\pi }{2}w_{k}^{2}\right)$, and $s_{k}=\sin\;\left(\frac{\pi }{2}w_{k}^{2}\right)$. Combining with Equation (A23) yields the derivatives used to establish Equation (31).

## Appendix E. Bopp Shifts for Hamiltonians Linear in Momentum in 3D

In three dimensions, let $\left(x,p\right)\in R^{3}\times R^{3}$ and let W(x,p,t) be a (Weyl) phase space density. We use the standard Weyl–Moyal ⋆-product written in Bopp-shift form:

(A28) $$\left(F⋆G\right)\left(x,p\right)=F\;\left(x+\frac{i\hbar }{2}\nabla_{\vec{p}},\;p-\frac{i\hbar }{2}\nabla_{\vec{x}}\right)G\left(x,p\right),$$

where $\nabla_{\vec{x}}$ and $\nabla_{\vec{p}}$ act on *G*. Equivalently,

(A29) $$\left(F⋆G\right)\left(x,p\right)=F\left(x,p\right)\exp\;\left[\frac{i\hbar }{2}\left({\overset{\leftarrow }{\nabla }}_{\;x}\cdot {\vec{\nabla }}_{\;p}-{\overset{\leftarrow }{\nabla }}_{\;p}\cdot {\vec{\nabla }}_{\;x}\right)\right]G\left(x,p\right).$$

Consider a Hamiltonian (Weyl symbol) that is linear in momentum,

(A30) H(x,p,t)=u(x,t)·p,

with u(x,t) independent of p. We compute the ⋆-commutator using (A28). First,

(A31) $$\left(H⋆W\right)\left(x,p\right)=u\;\left(x+\frac{i\hbar }{2}\nabla_{\vec{p}},t\right)\cdot \left(p-\frac{i\hbar }{2}\nabla_{\vec{x}}\right)W\left(x,p,t\right).$$

Since u depends only on x, its Bopp shift expands as

(A32) $$u\;\left(x+\frac{i\hbar }{2}\nabla_{\vec{p}},t\right)=u\left(x,t\right)+\frac{i\hbar }{2}\;\left(\nabla_{\vec{x}}u\right)\left(x,t\right)\;\nabla_{\vec{p}},$$

and the expansion truncates exactly at first order because *H* is linear in p. Substituting (A32) into (A31) and keeping the resulting terms gives

(A33) $$H⋆W=\left(u\cdot p\right)\;W-\frac{i\hbar }{2}\;u\cdot \nabla_{\vec{x}}W+\frac{i\hbar }{2}\;\left({\left(\nabla_{\vec{x}}u\right)}^{T}p\right)\cdot \nabla_{\vec{p}}W,$$

where ${\left(\nabla_{\vec{x}}u\right)}_{ij}=\partial_{x_{j}}u_{i}$ and ${\left({\left(\nabla_{\vec{x}}u\right)}^{T}p\right)}_{j}=\sum_{i}\left(\partial_{x_{j}}u_{i}\right)\;p_{i}$. Similarly,

(A34) $$\left(W⋆H\right)\left(x,p\right)=W\;\left(x-\frac{i\hbar }{2}\nabla_{\vec{p}},\;p+\frac{i\hbar }{2}\nabla_{\vec{x}}\right)\left(u\left(x,t\right)\cdot p\right),$$

which yields

(A35) $$W⋆H=\left(u\cdot p\right)\;W+\frac{i\hbar }{2}\;u\cdot \nabla_{\vec{x}}W-\frac{i\hbar }{2}\;\left({\left(\nabla_{\vec{x}}u\right)}^{T}p\right)\cdot \nabla_{\vec{p}}W.$$

Therefore, the ⋆-commutator is

(A36) $$\frac{1}{i\hbar }\;\left(H⋆W-W⋆H\right)=u\cdot \nabla_{\vec{x}}W-\left({\left(\nabla_{\vec{x}}u\right)}^{T}p\right)\cdot \nabla_{\vec{p}}W.$$

The right-hand side is exactly the Poisson bracket {H,W} in (x,p):

(A37) $$\left\{H,W\right\}=\left(\nabla_{\vec{p}}H\right)\cdot \left(\nabla_{\vec{x}}W\right)-\left(\nabla_{\vec{x}}H\right)\cdot \left(\nabla_{\vec{p}}W\right)=u\cdot \nabla_{\vec{x}}W-\left({\left(\nabla_{\vec{x}}u\right)}^{T}p\right)\cdot \nabla_{\vec{p}}W.$$

Hence, for Hamiltonians linear in momentum, the Moyal bracket coincides with the Poisson bracket *exactly* in 3D (and, in fact, in any dimension); all higher-order *ℏ*-corrections vanish because they require second (or higher) derivatives of *H* with respect to p.
